# Hybrid Cognitive Behavioral Therapy With Interoceptive Exposure for Irritable Bowel Syndrome: A Feasibility Study

**DOI:** 10.3389/fpsyt.2021.673939

**Published:** 2021-09-09

**Authors:** Misako Funaba, Hitomi Kawanishi, Yasushi Fujii, Koyo Higami, Yoshitoshi Tomita, Kazushi Maruo, Norio Sugawara, Yuki Oe, Satsuki Kura, Masaru Horikoshi, Chisato Ohara, Hiroe Kikuchi, Hajime Ariga, Shin Fukudo, Atsushi Sekiguchi, Tetsuya Ando

**Affiliations:** ^1^Department of Behavioral Medicine, National Institute of Mental Health, National Center of Neurology and Psychiatry, Kodaira, Japan; ^2^Department of Behavioral Medicine, Tohoku University Graduate School of Medicine, Sendai, Japan; ^3^Department of Psychology, Meisei University, Hino, Japan; ^4^Shinjuku-Gyoenmae Counseling Center, Tokyo, Japan; ^5^Department of Psychosomatic Medicine, National Center Hospital, National Center of Neurology and Psychiatry, Kodaira, Japan; ^6^Department of Biostatistics, Faculty of Medicine, University of Tsukuba, Tsukuba, Japan; ^7^Department of Psychiatry, Dokkyo Medical University School of Medicine, Mibu, Japan; ^8^Department of Neuropsychiatry, Kyorin University School of Medicine, Mitaka, Japan; ^9^National Center for Cognitive Behavioral Therapy and Research, National Center of Neurology and Psychiatry, Kodaira, Japan; ^10^Hidaka Dental Clinic, Fukuoka, Japan; ^11^Department of Clinical Psychology, Faculty of Human Sciences, Bunkyo University, Koshigaya, Japan; ^12^Department of Psychosomatic Medicine, Center Hospital of the National Center for Global Health and Medicine, Tokyo, Japan; ^13^Division of Gastroenterology, Department of General Internal Medicine, National Center Hospital, National Center of Neurology and Psychiatry, Kodaira, Japan; ^14^Department of Psychosomatic Medicine, Narita Hospital, International University of Health and Welfare, Narita, Japan

**Keywords:** feasibility study, complementary video materials, cognitive behavioral therapy with interoceptive exposure, irritable bowel syndrome, hybrid CBT

## Abstract

Irritable bowel syndrome (IBS) is a functional gastrointestinal disorder, which severely impairs the quality of life of patients. Treatment of refractory IBS patients is needed, but it is not yet widely available. Therefore, we previously developed a Japanese version of cognitive behavioral therapy with interoceptive exposure (CBT-IE) involving 10 face-to-face sessions to treat refractory IBS patients. To disseminate this treatment of IBS in places where therapists are limited, we further developed a hybrid CBT-IE program with complementary video materials that include psychoeducation and homework instructions so that patients can prepare for face-to-face sessions in advance at home and the session time can be shortened, thereby reducing the burden on both patient and therapist. In this study, we conducted a trial to evaluate the feasibility, efficacy, and safety of the hybrid CBT-IE program for Japanese IBS patients. The study was a single-arm, open-label pilot clinical trial. A total of 16 IBS patients were included in the study and 14 patients completed the intervention, which consisted of 10 weekly individual hybrid CBT-IE sessions. We performed an intention to treat analysis. The primary outcome measure for the efficacy of the intervention was a decrease in the severity of IBS symptoms. The feasibility and safety of the intervention were examined by the dropout rate and recording of adverse events, respectively. The dropout rate of the hybrid CBT-IE was comparable to that of our previous CBT-IE with only face-to-face sessions and no adverse events were recorded. The severity of IBS symptoms within-group was significantly decreased from the baseline to mid-treatment [Hedges' g = −0.98 (−1.54, −0.41)], post-treatment [Hedges' g = −1.48 (−2.09, −0.88)], 3-month follow-up [Hedges' g = −1.78 (−2.41, −1.14)], and 6-month follow-up [Hedges' g = −1.76 (−2.39, −1.13)]. Our results suggest that the hybrid CBT-IE is effective and could be conducted safely. To confirm the effectiveness of the hybrid CBT-IE, it is necessary to conduct a multicenter, parallel-design randomized control trial.

**Clinical Trial Registration:** [https://upload.umin.ac.jp/cgi-open-bin/ctr/ctr_view.cgi?recptno=R000041376], identifier [UMIN000036327].

## Introduction

Irritable bowel syndrome (IBS) is a disorder of brain-gut interactions characterized by abdominal pain and bowel movement problems, such as diarrhea and constipation (1). Although IBS is not a fatal disease, patients' quality of life (QOL) can be significantly impaired (2, 3). Approximately 4.1% of the population worldwide are reported to be affected by IBS symptoms (4). The core pathophysiology of IBS is hypersensitivity to visceral stimulation involving increased autonomic arousal to visceral events (5, 6). Clinical and neurological studies have suggested that elevated central stress response enhances visceral sensitivity (7), which is similar to interoceptive hypersensitivity (8, 9). Gastrointestinal symptom-specific anxiety may play an important role in increasing pain sensitivity, hypervigilance, and poor coping behaviors (10, 11). As a result, visceral anxiety has been considered as the primary affective disturbance in IBS and as the mediator between other risk factors (e.g., neuroticism, trait anxiety, and worry) and IBS symptom severity (12). IBS symptoms can worsen with stressful situations or stimuli (13). These phenomena imply reciprocal brain-gut interactions as the mechanism between the symptoms of IBS and psychological processes (14).

Cognitive behavioral therapy (CBT) is applied not only to mental illness but also to refractory IBS. In the clinical guidelines for IBS proposed by the Japanese Society of Gastroenterology, the treatment of IBS consists of three stages (15, 16). If the patients' condition does not improve in the first stage (e.g., diet, life-style guidance, and pharmacotherapies for gastrointestinal symptoms), the second stage (e.g., psychotropic drug therapy) is applied. Finally, psychotherapy (e.g., CBT, hypnotherapy, and relaxation methods) is used as the third stage of intervention for refractory IBS (17)^1^. Among the various types of CBT protocols, CBT using interoceptive exposure (CBT-IE) to visceral sensations is one of the most promising psychotherapies for IBS. Interoceptive exposure (IE) focuses on reducing anxiety and avoidance response to visceral sensations. IE weakens the fear response by enabling new learning that competes with the initial fearful associations (18). CBT-IE consists of two components. The first is similar to traditional CBT and includes education about IBS symptoms that reflect conditional reactions to reminders of gastrointestinal distress, self-monitoring of IBS symptoms, attention control training to learn to shift focus away rather than perseverate unpleasant visceral sensations (19), cognitive therapy to identify and challenge threat-laden appraisals of visceral sensations, and *in-vivo* exposure to feared/avoided situations. The second component is IE with repeated exposure to visceral sensations, such as tightening the abdomen to produce gut sensations, delaying defecation, and eating feared/avoided foods. IE is expected to reduce fear of sensations, as the procedure is aimed at alleviating gastrointestinal symptom-specific anxiety by purposely evoking bodily sensations that IBS patients fear (18). Craske et al. examined the efficacy of CBT-IE protocol compared to stress management (SM) or attention control (AC). They reported that CBT-IE outperformed AC on several indexes of outcome, and outperformed SM in some domains. Incidentally, no differences were observed between SM and AC. The results suggest that CBT-IE may be a particularly effective treatment for IBS (18).

Therefore, we developed a Japanese version of CBT-IE involving 10 face-to-face sessions including the same contents as the original CBT-IE for IBS (18). Our feasibility study of the Japanese version of CBT-IE showed a significant reduction of IBS symptoms and a remarkable improvement in IBS-specific QOL post-intervention, at the 3-month and 6-month follow-ups, compared with the pre-intervention state (20). Originally, the Japanese version of face-to-face CBT-IE (20) consisted of 10 × 60-min sessions. We realized that this structure was burdensome for patients and therapists and disadvantageous for widespread use in the current situation where the number of therapists is limited, and this led us to develop a hybrid CBT-IE. To overcome the difficulties in disseminating this intervention widely to clinical settings in Japan, due to a shortage of cognitive behavioral therapists and the highly time-consuming process of CBT (20), we further developed a hybrid CBT-IE program. This comprised complementary video materials, including psychoeducation and homework instructions, to allow patients to prepare at home before each face-to-face session. Consequently, the length of the face-to-face sessions was shortened from 60 to 30 min.

In this study, we conducted a trial to evaluate the feasibility, efficacy, and safety of the hybrid CBT-IE program for Japanese IBS patients. The efficacy of the intervention was measured by whether a significant reduction of IBS symptoms was reported post-treatment during follow-ups compared with the pre-intervention. Feasibility and safety were evaluated by the dropout rate and incidence of severe adverse events, respectively.

We hypothesized that severity of IBS, abdominal anxiety, IBS-related QOL and health-related QOL would improve at the end of the hybrid CBT-IE as well as at follow-ups compared with the baseline.

## Methods

### Study Design

This study was a single-arm, open-label trial. The trial was registered as a feasibility study and conducted at the National Center of Neurology and Psychiatry (NCNP) Hospital in Kodaira, Japan.

### Participants and Recruitment

Participants were recruited from an IBS-specialized outpatient unit of the NCNP Hospital; they were referred by their primary physicians or voluntarily contacted the researchers in response to an advertisement on the NCNP homepage.

**Figure 1:**
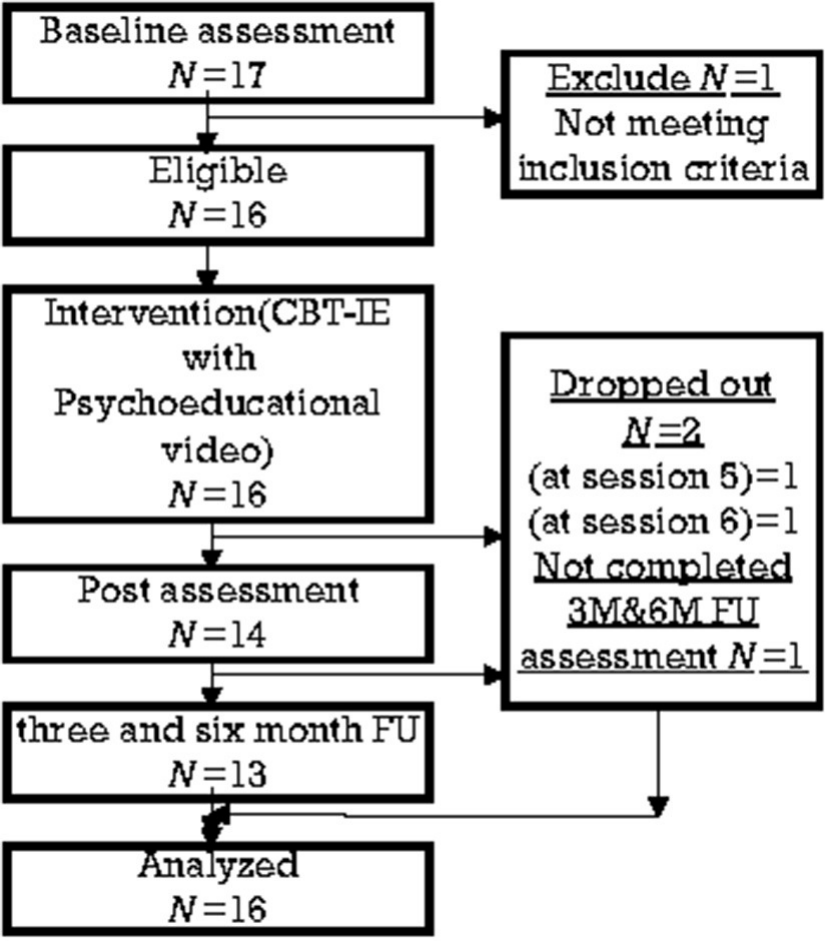
Diagram.

The flow of participants is shown in Figure 1. Participants were included if they (i) were diagnosed by physicians (TA, YT, and HA) as suffering IBS according to the Rome III criteria (21); (ii) were at least 16 years old at the time of screening assessment; (iii) showed ≥175 points (i.e., moderate severity) on the Irritable Bowel Syndrome Severity Index (IBSSI-J) during screening assessment; and (iv) were able to understand the purpose of this study and its contents, and provide written informed consent. Participants could withdraw at any time without penalty. The Mini-International Neuropsychiatric Interview (M.I.N.I.) was also conducted as screening by physicians (TA or YT). The subjects were assessed through interviews with the researchers, two psychosomatic physicians, and the subjects were included in the study. The exclusion criteria are shown in Table 1.

**Table 1:** Eligibility criteria and warning symptoms list.

1. Person in whom organic disease is suggested by the presence of warning symptoms*.
2. Persons with a history of or concomitant inflammatory bowel disease, malignant tumor, or other bowel disease which could cause the current bowel symptoms.
3. Persons suffering from major psychiatric disease, such as psychotic disorders, bipolar disorder, substance abuse-related disorders, or eating disorders (persons with anxiety disorders and depression without suicidal ideation are not excluded)
4. Persons with antisocial personality disorders.
5. Persons observed to have significant suicidal ideation at screening.
6. Persons with another past or present psychiatric or physical disease that is likely to interfere with continuation and evaluation of the study.
7. Persons experiencing any other type of marked chronic pain.
8. Those taking narcotic analgesics.
9. Persons who anticipate difficulty attending 10 sessions as an outpatient during the 16-week CBT implementation period.
10. Those who have previously received structured individual CBT.
11. Those for whom verbal and written communication in Japanese is not possible.
12. Pregnant or lactating women.
13. Any other person whom the principal investigator has determined to be unsuitable as a participant of the study.
*Warning symptoms list.
1. Symptoms which first appeared after 50 years of age.
2. Any rectal bleeding that has not undergone sufficient medical investigation (excluding that caused by known hemorrhoids).
3. Diarrhea-predominant IBS in which no colonoscopy investigation has been conducted.
4. Unexplained weight loss without a change in eating habits.
5. Nocturnal symptoms sufficient to cause insomnia.
6. The presence of warning symptoms (anemia, inflammatory reactions, or fecal occult blood).
7. Persons with a family history of colon cancer in a first- or second-degree relative (grandparents, parents, siblings, or children).

*https://upload.umin.ac.jp/cgi-open-bin/ctr/ctr_view.cgi?recptno=R000041376*.

### Sample Size

We determined a target sample size of 20 participants considering dropout rates (15%) estimated based on our previous face-to-face feasibility study of CBT-IE for IBS (20) and the recommendation that more than 12 participants are suitable for pilot studies (22).

### Study Procedures

After the screening, the hybrid CBT-IE interventions were conducted at the outpatient service in the NCNP Hospital for eligible IBS participants. Participants completed a baseline assessment before the first session, a mid-treatment assessment when they finished their fifth session of CBT-IE, and a post-treatment assessment when all sessions were completed. At 3 and again at 6 months after completion of the intervention, a follow-up assessment was performed.

### Hybrid CBT-IE for IBS

**Figure 2:**
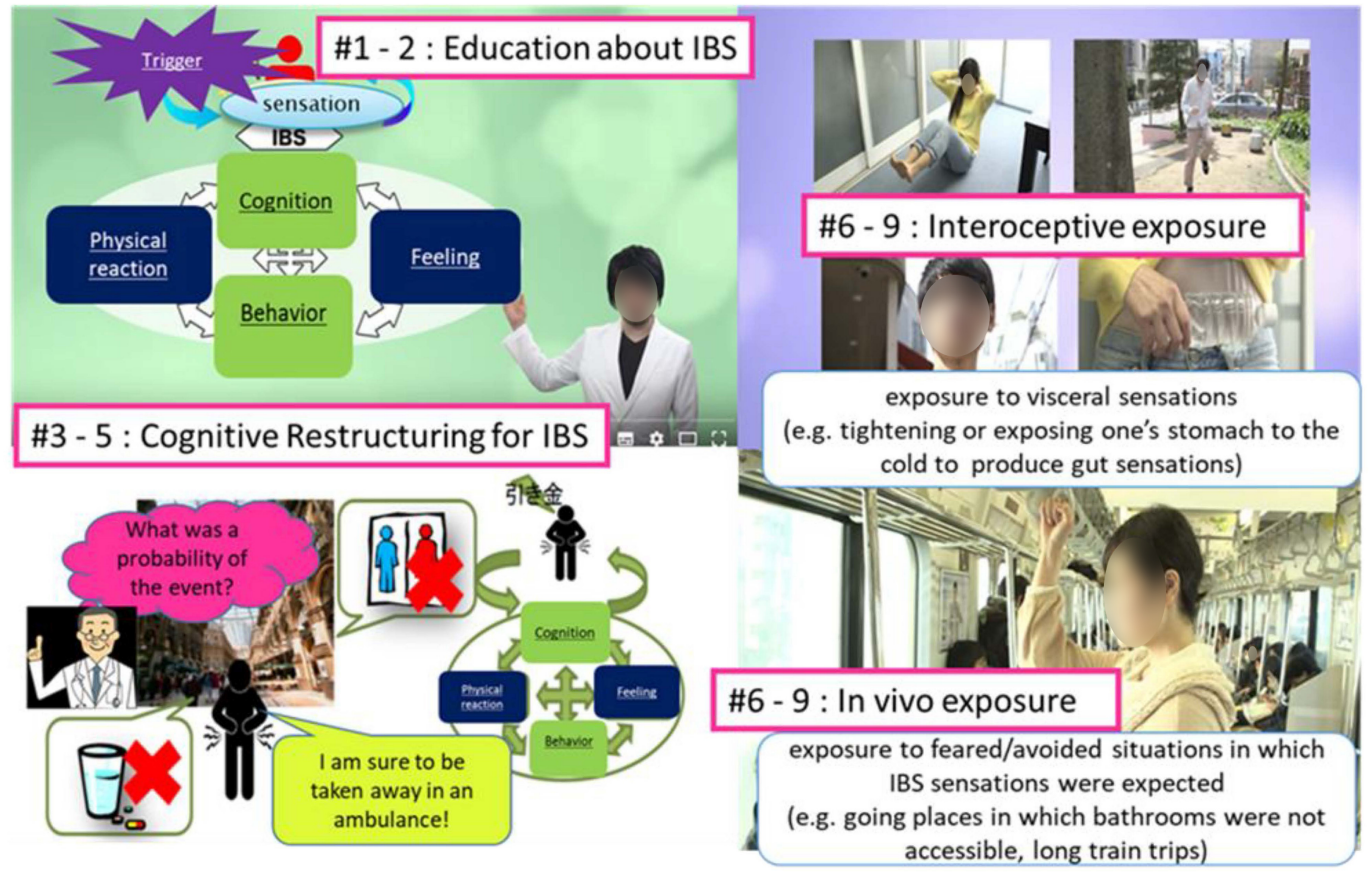
Contents of psychoeducational videos.

We developed a hybrid CBT-IE protocol for this study consisting of face-to-face sessions and self-study using psychoeducational videos based on Craske et al.'s original CBT-IE (18). We did not change the contents of the original protocol, except for making a textbook and psychoeducational video materials for patients. The textbook contents included pictures, illustrations, and figures to aid patients' understanding. It also contained homework worksheets for self-monitoring. The video materials consisted of 10 lectures about IBS mechanisms and behavioral-cognitive skills. CBT-IE consisted of the following seven components: (1) psychoeducation about IBS symptoms, including the mechanism by which symptoms are maintained; (2) self-monitoring and development of the CBT model of IBS; (3) learning AC skills for modifying attention bias to visceral sensations; AC (19) is training that teaches patients to shift the focus of unpleasant visceral sensations, rather than tolerate them. In this program, multiple sounds, such as a metronome and noise, are presented simultaneously, and voice guidance is used to practice paying attention to and switching between each sound; (4) cognitive restructuring for the anxiety related to IBS symptoms and visceral sensations; (5) *in-vivo* exposure to situations that each patient feared or avoided because of anxiety about the occurrence of IBS symptoms (which was personalized considering each participant's tolerance); (6) IE to abdominal sensations that patients feared, for example, by tightening a belt on patients' midriff or drinking something cold; and (7) relapse prevention (see Table 2, Figure 2).

**Table 2:** Contents of each session (23).

**Session number**	**Contents of intervention**	**Handouts**	**Play time of Video**
1	Education about IBS and psychological stress on digestive functioning, awareness-raising	• Personal IBS profile (in session use) • Monitoring IBS distress	(12′ 16″)
2	Education about the role of conditioning in IBS, attentional training	• Monitoring IBS distress • Guide for Attentional training • Common IBS symptom appraisal list	(6′ 44″)
3	Attentional training, cognitive restructuring for IBS sensations and risk estimates	• Monitoring IBS distress • Common IBS symptom appraisal list	(9′ 31″)
4	Cognitive restructuring for symptoms of IBS, valence estimates, hierarchy construction for IBS sensation reminders	• Monitoring IBS distress • Deliberate exposure hierarchy	(9′ 05″)
5	Cognitive restructuring, interoceptive exposure assessment, *in vivo* exposure	• Monitoring IBS distress • Interoceptive exposure exercises • Interoceptive exposure FAQ • Guide for IBS and *in-vivo* exposure • *In vivo* exposure instructions • Deliberate exposure record	(11′ 36″)
6–9	Conduct of Interoceptive exposure, *in vivo* exposure	• Monitoring IBS distress • Interoceptive exposure instructions • Interoceptive exposure record	(5′ 40″) (3′ 32″) (3′ 43″) (3′ 35″)
10	Interoceptive exposure, *in vivo* exposure, summary of the all sessions, relapse prevention	• Monitoring IBS distress • Relapse prevention Map • Dealing with setbacks • List of positive Accomplishments	(7′ 44″)

Patients were offered 10 face-to-face session of 30 min each combined with pre-learning of video material (Figure 2). One course was performed within 4 months. Before the face-to-face session began, we gave the subjects the printed material and YouTube URL for the first session. At the end of each session, we gave the subjects both the YouTube URL and video material for the next session. Participants were instructed to recognize IBS symptoms and apply cognitive behavioral skills through the video as homework before each session. Patients' understanding of the contents of the video and text, and their practice of homework was reviewed in the subsequent face-to-face session.

### Therapists

The hybrid CBT-IE interventions were conducted by two clinical psychologists (HitK and MF) with clinical experience in a psychiatric setting of 7 and 18 years, respectively. Interventions were supervised by the third author (YF), who is a licensed clinical psychologist, and the last author (TA), who is a specialist in psychosomatic medicine. Medical treatments and management were performed by two physicians who are specialists in psychosomatic medicine (TA and YT).

### Quality Assurance

Therapists used a therapeutic manual of CBT-IE to practice all seven components. They also received regular training on hybrid CBT-IE by supervisors (TA and YF) to maintain treatment fidelity. All the hybrid CBT-IE sessions were subject to evaluations of treatment adherence using treatment manuals.

### Homework Adherence

Therapists asked participants certain questions to check for homework adherence. Examples of questions are as follows: “Did you watch the video before the session?” “Please explain what you understand about the video,” and “Are there any questions about this session?” When participants asked questions about the session, the therapist provided details or gave tips about doing homework. If a participant did not do the homework, the therapist explained the session's content and worked on the homework with the participant.

### Safety Monitoring

In this study, subjects were asked to report adverse events at every CBT session and physician visit. Each participant received checkups every 2 weeks or once a month and adverse events were examined by physicians (TA and YT) throughout the intervention period. The therapists also monitored severe adverse events at every CBT-IE session from participants' verbal reports.

### Medication

Each participant's unique medication was kept constant or could be reduced, but neither increased doses or new doses were allowed throughout the research period.

### Measures

#### Primary Outcome Measure: IBS Severity Index

The Japanese version of the IBS Severity Index (IBSSI-J, which is the same as the Japanese version of the IBS Symptoms Severity Scale, IBS-SSS) evaluates the severity of IBS symptoms and is a valid and reliable assessment tool for Japanese patients (24, 25). This self-reported instrument has items that score abdominal pain, abdominal distention, bowel movements, and QOL. The total score ranges from 0 to 500. Severity is graded as mild (75–174), moderate (175–299), or severe (300–500).

#### Secondary Outcome Measures

##### Gastrointestinal Symptom-Specific Anxiety (Visceral Sensitivity Index)

The Visceral Sensitivity Index (VSI) scale evaluates gastrointestinal symptom-specific anxiety (10, 26). The scale includes 15 items scored on a 6-point Likert scale. Lower scores indicate greater severity of anxiety about abdominal symptoms.

##### Disease-Specific Quality of Life for IBS

The disease-specific quality of life for IBS (IBS-QOL) measure was used to assess IBS-specific QOL (27). This 34-item 5-point Likert scale examines how IBS affects the daily functioning of a participant. The scale includes eight subscales: dysphoria, interference with activity, body image, health worry, food avoidance, social reaction, sexual concerns, and relationships. A higher total score of subscales combined indicates better QOL.

##### Health-Related QOL (36-Item Short-Form Health Survey)

The health-related QOL was assessed using the short-form health survey (SF-36) (28). This 36-item scale consists of eight subscales: physical functioning, physical role, bodily pain, general health, vitality, social functioning, emotional role, and mental health. A higher total score indicates better health related QOL.

##### Anxiety (State-Trait Anxiety Inventory)

Anxiety was assessed using the State-Trait Anxiety Inventory (STAI) (29). This 40-item scale, answered using 4-point Likert scales, assesses both trait and state anxiety. A higher total score indicates the presence of higher intensity anxiety.

##### Depression (Beck Depression Inventory-II)

Depression was assessed using the Beck Depression Inventory-II (BDI-II) (30, 31). This 21-item scale utilizes a 4-point Likert scale, and a higher total score indicates the presence of higher severity depression.

##### Irritable Bowel Syndrome Global Improvement Scale

In the IBS Global Improvement Scale (IBS-GIS), patients assess improvement of IBS using a 7-point Likert scale (32). Participants completed a questionnaire mid-treatment, post-treatment, and at the 3-month and 6-month follow-ups by recording a rating of IBS global improvement (“Compared to the way you usually felt during the 3 months before you entered the study, have your IBS symptoms over the past 4 weeks been substantially worse = 7, moderately worse = 6, slightly worse = 5, unchanged = 4, slightly improved = 3, moderately improved = 2, or substantially improved = 1?”). In line with previous research (32), we defined people with a score less than 3 (with 1 or 2) on the IBS-GIS as treatment responders in this study.

### Ethical Approval

Prior to the start of this study, participants provided written informed consent. This study received approval from the ethical review board of the NCNP (approval number: A2015-118). The study was also registered to a clinical trials registry (UMIN000036327; https://upload.umin.ac.jp/cgi-open-bin/ctr/ctr_view.cgi?recptno=R000041376).

### Data Analysis

We calculated the dropout rate to evaluate the feasibility of the hybrid CBT-IE for IBS. In addition, we estimated the change in all outcome variables over time based on a linear mixed model (LMM) considering missing values due to dropouts with SPSS version 26 (SPSS Inc) with intention to treat. In this analysis, each assessment period was included as a categorical fixed effect and participants were included as a random effect. The LMM can be applied to test the difference of means between conditions for data that have been measured repeatedly under several conditions. In the LMM analysis, the assessment period (level: pre-assessments, mid-assessments, and post-assessments) was included as the categorical fixed effect and participants were included as a random effect. Then we estimated treatment effect for each assessment period based on parameter estimates of fixed effects. Thus, we reduced the bias caused by missing values due to dropout, compared to the case where only the data at each time point are used for estimation. In addition, Hedges' g showed a 95% confidence interval from the LMM for each treatment visit (pre-mid treatment, pre-post treatment, pre-3 month follow up, pre-6 month follow up, post-3 month follow up, and post-6 month follow up) within-group was calculated to examine the impact of the hybrid CBT-IE's efficacy using an effect size calculator (https://www.cem.org/effect-size-calculator).

As a supplementary analysis, we compared the dropout and responder rates at post-treatment and follow-ups using 50% or greater improvement on the IBSSI-J from the baseline and VSI, the IBS-GIS as defined in the GIS section of the face-to-face only CBT-IE, and the hybrid CBT-IE feasibility study using the chi-square test.

## Results

### Characteristics of Participants

The recruitment of participants began in October 2016 and the last 6-month follow-up assessment ended in November 2019. Table 3 indicates the characteristics of participants. A total of 17 participants were screened and 16 of them were eligible. One participant was ruled out by a doctor because a comorbid olfactory reference syndrome, which was thought to interfere with the implementation of CBT-IE, was identified after screening. Two participants dropped out before they completed the intervention. One participant lost motivation before the fifth session. Another dropped out before the sixth session, getting depressed by the ongoing distress of a long-standing family conflict.

**Table 3:** Demographic data.

**Demographic variables**	**Value**
**Gender**	
Women, *N* (%)	12 (71)
**Age (years), mean (** ***SD*** **)**	36.76 (13.41)
Median	35
Range	17–65
**Duration of IBS (years), mean (** ***SD*** **)**	14.12 (11.66)
Median	9
Range	2–45
**Type of IBS**, ***N*****(%)**	
IBS-D	13 (76)
IBS-C	1 (6)
IBS-M	0 (0)
IBS-U	3 (18)
**Employment status**, ***N*****(%)**	
Employed full-time	7 (41)
Employed full-time, suspended from work	1 (6)
Employed part-time	1 (6)
Unemployed	8 (47)
**Marital status**, ***N*****(%)**	
Single	7 (41)
Married	9 (53)
Divorce/Widow	1 (6)
**Educational background**, ***N*****(%)**	
High school student	1 (6)
High school	2 (12)
≧2 years of college/university	14 (82)

The median IBS duration of participants included this study was 9 years (range of 2–45 years). The percentage of IBS types were: IBS with diarrhea (IBS-D) = 76%, IBS with constipation (IBS-C) = 6%, mixed IBS (IBS-M) = 0%, and unclassified IBS (IBS-U) = 18%.

### Comorbidities

One participant had comorbid panic disorder with agoraphobia and social anxiety disorder (6%), another participant had comorbid agoraphobia and general anxiety disorder (6%), and three participants had comorbid agoraphobia (18%) based on the M.I.N.I (Table 3).

### Dropout Rates

The dropout rate in this study was 12.5% (*N* = 2/16), which is similar to the dropout rate of our previous feasibility study of CBT-IE for IBS with face-to-face sessions (15%, *N* = 3/20) (20). A chi-square test showed no statistically significant differences between the two.

### Primary Outcome Measures

Table 4 shows the mean and standard deviation of the primary and secondary outcome measures at baseline, mid-treatment, post-treatment, and 3- and 6-month follow-up assessments. Table 5 shows estimated mean differences (MD) and standardized mean differences (Hedges' g) of the outcome measures with a 95% confidence interval. The *post-hoc* power for the primary endpoint at post treatment visit was estimated as >99%.

**Table 4:** Descriptive statistics of outcome measures (mean and standardized deviation).

	**Pre**		**Mid**		**Post**		**3 months follow up**		**6 months follow up**	
	**Mean**	***SD***	**Mean**	***SD***	**Mean**	***SD***	**Mean**	***SD***	**Mean**	***SD***
**IBSSI-J**	294.06	75.79	206.43	71.88	165.00	88.90	137.88	77.51	139.23	82.26
**VSI**	19.94	14.18	30.21	13.74	37.79	12.22	41.15	16.12	41.08	18.63
**IBS-QOL**										
Tolal	52.63	18.87	70.29	19.06	81.00	9.49	82.00	11.30	80.69	12.87
Dysphoria	38.50	22.00	59.57	27.65	75.71	16.34	80.31	10.87	77.38	16.90
Interference with activity	38.44	21.81	56.00	24.27	70.00	13.50	71.69	16.22	72.38	16.16
Body image	75.94	22.52	85.43	19.54	89.43	10.78	88.08	15.80	88.08	12.26
Health worry	50.94	25.55	81.57	22.13	86.36	16.60	86.00	14.62	82.69	16.87
Food avoidance	43.19	37.28	67.79	28.82	84.50	15.27	81.46	21.53	79.46	18.73
Social reaction	74.00	18.45	83.57	16.78	91.21	9.67	88.62	13.27	88.62	12.65
Sexual concerns	86.00	18.71	93.79	16.00	96.43	9.08	94.62	9.49	95.31	10.69
Relationships	53.56	20.84	70.93	18.47	76.71	15.72	80.38	12.65	78.92	18.26
**SF-36**										
Physical functioning	49.75	15.11	54.50	5.83	53.93	5.57	56.00	2.80	59.54	11.70
Physical role	46.06	10.58	48.21	9.64	49.79	7.90	51.00	5.29	56.31	12.98
Bodily pain	44.19	13.47	44.00	14.67	49.79	9.79	50.31	9.12	53.54	13.63
General health	40.81	12.83	42.29	9.38	48.50	12.13	47.92	12.18	48.92	19.01
Vitality	44.06	11.19	47.29	7.19	48.29	9.84	46.23	11.94	50.25	19.82
Social functioning	46.94	14.83	47.79	11.54	53.36	7.86	51.62	6.85	52.58	17.14
Emotional role	44.69	10.58	50.36	7.83	50.71	8.05	52.23	5.95	55.08	14.34
Mental health	44.06	9.15	47.86	6.87	50.79	7.23	49.00	7.89	51.83	15.47
**STAI**										
Trait anxiety	53.69	10.22	49.36	9.06	45.86	10.06	46.46	11.38	45.15	12.19
State anxiety	47.31	11.25	39.93	10.82	38.00	8.68	38.77	10.93	37.85	9.86
**BDI-II**										
Total	12.31	6.71	8.29	7.47	6.57	5.50	6.85	7.29	6.85	6.41

*IBSSI, Irritable Bowel Syndrome Severity Index; VSI, Vischeral Sensitivity Index; IBS-QOL, Irritable Bowel Syndrome-Quality Of Life; SF-36, MOS 36-Item Short-Form Health Survey; STAI, State-Trait Anxiety Inventory; BDI-II, Beck Depression Inventory-II*.

**Table 5:** Estimated mean difference and standardized mean difference with 95% confidence interval LMM.

	**Baseline to mid-treatment**		**Baseline to post-treatment**		**Baseline to 3** **months follow up**		**Baseline to 6** **months follow up**		**Post-treatment to 3** **months follow up**		**Post-treatment to 6** **months follow up**	
	**MD (95%CI)**	**SMD**	**MD (95%CI)**	**SMD**	**MD (95%CI)**	**SMD**	**MD (95%CI)**	**SMD**	**MD (95%CI)**	**SMD**	**MD (95%CI)**	**SMD**
		**(Hedges'g, 95%CI)**		**(Hedges'g, 95%CI)**	**(Hedges'g, 95%CI)**		**(Hedges'g, 95%CI)**	**(Hedges'g, 95%CI)**		**(Hedges'g, 95%CI)**
**IBSSI-J total**	**−79.80**	**−0.98**	**−121.22**	**−1.48**	**−144.96**	**−1.78**	**−143.61**	**−1.76**	−23.73	−0.29	−22.34	−0.27
**(primary measure)**	**[−132.18**, **−27.40]**	**[−1.54**, **−0.41]**	**[−173.61**, **−68.84]**	**[−2.09**, **−0.88]**	**[−198.63**, **−91.28]**	**[−2.41**, **−1.14]**	**[−197.28**, **−89.94]**	**[−2.39**, **−1.13]**	[−85.50, 38.04]	[−0.84, 0.26]	[−84.15, 39.38]	[−0.82, 0.27]
**VSI total**	**8.79 [0.99, 16.60]**	**0.57 [0.02, 1.12]**	**16.36 [8.56, 24.17]**	**1.06 [0.49, 1.63]**	**18.51 [10.51,26.51]**	**1.20 [0.62, 1.78]**	**18.43 [10.43, 26.43]**	**1.19 [0.61, 1.78]**	2.14 [−7.01, 11.30]	0.14 [−0.40, 0.68]	2.07 [−7.09, 11.22]	0.13 [−0.41, 0.68]
**IBS-QOL**												
Tolal	**16.05 [6.78, 25.33]**	**1.04 [0.46, 1.61]**	**26.77 [17.49, 36.05]**	**1.73 [1.1, 2.36]**	**27.15 [6.78, 25.33]**	**1.75 [1.12, 2.38]**	**25.84 [16.33, 35.35]**	**1.67 [1.05, 2.29]**	0.38 [−10.54, 11.30]	0.02 [−0.52, 0.57]	−0.93 [−11.85, 9.99]	−0.06 [−0.60, 0.48]
Dysphoria	**19.75 [6.16, 33.34]**	**0.96 [0.40, 1.53]**	**35.90 [22.31, 49.49]**	**1.75 [1.12, 2.38]**	**38.57 [24.65, 52.50]**	**1.88 [1.24, 2.52]**	**35.65 [21.73, 49.57]**	**1.74 [1.11, 2.36]**	2.68 [−13.40, 18.72]	0.13 [−0.41, 0.68]	−0.25 [−16.28, 15.79]	−0.01 [−0.56, 0.53]
Interference with activity	**16.07 [4.68, 27.46]**	**0.83 [0.27, 1.39]**	**30.07 [18.68, 41.46]**	**1.55 [0.95, 2.16]**	**30.66 [18.99, 42.34]**	**1.59 [0.97, 2.20]**	**31.35 [19.68, 43.02]**	**1.62 [1.01, 2.24]**	0.59 [−12.81, 14.68]	0.03 [−0.51, 0.57]	1.28 [−12.12, 14.68]	0.07 [−0.48, 0.61]
Body image	8.03 [−0.31, 16.38]	0.45 [−0.09, 0.99]	**12.03 [3.69, 20.38]**	**0.68 [0.12, 1.23]**	**11.78 [3.22, 20.33]**	**0.66 [0.11, 1.21]**	**11.78 [3.22, 20.33]**	**0.66 [0.11, 1.21]**	−0.26 [−10.03, 9.52]	−0.01 [−0.56, 0.53]	−0.26 [−10.03, 9.52]	−0.01 [−0.56, 0.53]
Health worry	**29.79 [18.28, 41.30]**	**1.50 [0.90, 2.10]**	**34.57 [23.06, 46.08]**	**1.74 [1.12, 2.37]**	**33.59 [21.79, 45.39]**	**1.69 [1.08, 2.31]**	**30.28 [18.49, 42.08]**	**1.53 [0.92, 2.13]**	−0.98 [−14.52, 12.55]	−0.05 [−0.59, 0.49]	−4.29 [−17.82, 9.24]	−0.22 [−0.76, 0.32]
Food avoidance	**21.17 [4.96, 37.38]**	**0.77 [0.21, 1.33]**	**37.88 [21.67, 54.09]**	**1.39 [0.78, 1.99]**	**33.93 [17.32, 50.54]**	**1.24 [0.65, 1.83]**	**31.93 [15.32, 48.54]**	**1.17 [0.58, 1.75]**	−3.95 [−23.03, 15.12]	−0.14 [−0.69, 0.40]	−5.91 [−25.03, 13.12]	−0.22 [−0.77, 0.33]
Social reaction	**8.43 [0.53, 16.32]**	**0.56 [0.01, 1.11]**	**16.07 [8.18, 23.97]**	**1.07 [0.50, 1.64]**	**14.04 [5.95, 22.14]**	**0.94 [0.37, 1.50]**	**14.04 [5.95, 22.14]**	**0.94 [0.37, 1.50]**	−2.03 [−11.29, 7.24]	−0.13 [−0.68, 0.41]	−2.03 [−11.29, 7.24]	−0.13 [−0.68, 0.41]
Sexual concerns	5.69 [−4.23, 15.60]	0.39 [−0.18, 0.95]	8.33[−1.58, 18.24]	**0.57 [0.00, 1.13]**	6.89 [−3.26, 17.05]	0.47 [−0.10, 1.03]	7.58 [−2.57, 17.74]	0.51 [−0.05, 1.08]	−1.44 [−13.16, 10.28]	−0.10 [−0.67, 0.47]	−0.75 [−12.47, 10.98]	−0.05 [−0.62, 0.52]
Relationships	**15.72 [4.44, 27.00]**	**0.88 [0.32, 1.44]**	**21.50 [10.22, 32.79]**	**1.20 [0.62, 1.78]**	**25.42 [13.86, 36.97]**	**1.42 [0.82, 2.02]**	**23.95 [12.40, 35.51]**	**1.34 [0.75, 1.93]**	3.91 [−9.38, 17.20]	0.22 [−0.33, 0.76]	2.45 [−10.84, 15.74]	0.14 [−0.41, 0.68]
**SF-36**												
Physical functioning	2.56 [−3.62, 8.73]	0.20 [−0.37, 0.77]	1.98 [−4.19, 8.16]	0.16 [−0.41, 0.72]	4.26 [−2.07, 10.59]	0.34 [−0.23, 0.91]	**7.80 [1.47, 14.12]**	**0.62 [0.04, 1.20]**	2.28 [−4.97, 9.52]	0.18 [−0.40, 0.76]	5.81 [−1.43, 13.06]	0.46 [−0.12, 1.04]
Role physical	1.90 [−6.09, 9.89]	**0.81 [0.25, 1.36]**	3.47 [−4.52, 11.47]	**0.97 [0.41, 1.53]**	4.71 [−3.47, 12.89]	**1.09 [0.53, 1.66]**	**10.02 [1.84, 18.20]**	**1.63 [1.02, 2.25]**	1.24 [−8.26, 10.74]	0.13 [−0.41, 0.67]	6.55 [−2.95, 16.04]	**0.67 [0.11, 1.22]**
Bodily pain	−0.84 [−11.21, 9.53]	−0.07 [−0.61, 0.47]	4.95 [−5.42, 15.32]	0.39 [−0.15, 0.93]	5.70 [−4.90, 16.31]	0.45 [−0.09, 0.99]	8.93 [−1.67, 19.54]	**0.70 [0.15, 1.26]**	0.75 [−11.57, 13.78]	0.06 [−0.49, 0.61]	3.98 [−8.34, 16.31]	0.31 [−0.24, 0.87]
General health	0.83 [−7.91, 9.56]	0.06 [−0.47, 0.59]	7.04 [−1.70, 15.78]	0.52 [−0.02, 1.06]	5.75 [−3.20, 14.70]	0.42 [−0.12, 0.96]	6.75 [−2.20, 15.70]	0.50 [−0.04, 1.04]	−1.29 [−11.59, 9.01]	−0.09 [−0.64, 0.45]	−0.29 [−10.59, 10.01]	−0.02 [−0.56, 0.52]
Vitality	2.77 [−5.06, 10.59]	−0.03 [−0.58, 0.51]	3.77 [−4.06, 11.59]	0.40 [−0.15, 0.95]	1.31 [−6.71, 9.33]	0.20 [−0.35, 0.75]	5.29 [−2.94, 13.52]	0.22 [−0.32, 0.77]	−2.46 [−11.67, 6.76]	−0.19 [−0.74, 0.36]	1.53 [−7.93, 10.98]	0.12 [−0.43, 0.67]
Social functioning	−0.41 [−9.35, 8.52]	−0.03 [−0.58, 0.52]	5.16 [−3.78, 14.09]	0.40 [−0.16, 0.96]	2.60 [−6.55, 11.75]	0.20 [−0.35, 0.75]	3.41 [−5.98, 12.80]	0.26 [−0.29, 0.82]	−2.56 [−13.12, 8.01]	−0.20 [−0.76, 0.37]	−1.75 [−12.58, 9.09]	−0.14 [−0.70, 0.43]
Role emotional	5.63 [−1.66, 12.92]	**0.57 [0.04, 1.11]**	5.99 [−1.30, 13.28]	**0.61 [0.07, 1.15]**	**7.53 [0.06, 14.99]**	**0.77 [0.23, 1.31]**	**10.37 [2.91, 17.83]**	**1.06 [0.50, 1.62]**	1.54 [−7.09, 10.16]	0.16 [−0.38, 0.69]	4.38 [−4.25, 13.01]	0.45 [−0.09, 0.99]
Mental health	3.59 [−3.15, 10.34]	0.37 [−0.17, 0.91]	6.52 [−0.22, 13.27]	**0.67 [0.12, 1.22]**	4.10 [−2.80, 11.01]	0.42 [−0.12, 0.96]	6.81 [−0.28, 13.90]	**0.70 [0.15, 1.25]**	−2.42[−10.39, 5.55]	−0.25 [−0.79, 0.30]	0.29 [−7.88, 8.46]	0.03 [−0.51, 0.57]
**STAI**												
Trait anxiety	−3.89 [−8.14, 0.37]	−0.38 [−0.92, 0.16]	**−7.39 [−11.64**, **−3.14]**	**−0.71 [−1.27**, **−0.16]**	**−6.34 [−10.70**, **−1.98]**	**−0.61[−1.16**, **−0.07]**	**−7.66 [−12.00**, **−3.29]**	**−0.74 [−1.29**, **−0.19]**	1.05 [−3.92, 6.02]	0.10 [−0.44, 0.64]	−0.26 [−5.23, 4.71]	−0.03 [−0.57, 0.52]
State anxiety	**−7.04 [−12.90**, **−1.17]**	**−0.67 [−1.21**, **−0.12]**	**−8.97 [−14.83**, **−3.10]**	**−0.85 [−1.41**, **−0.29]**	**−7.07 [−13.08**, **−1.06]**	**−0.67[−1.22**, **−0.12]**	**−8.00 [−14.01**, **−1.99]**	**−0.76 [−1.31**, **−0,21]**	1.89 [−5.00, 8.78]	0.18 [−0.36, 0.72]	0.97 [−5.92, 7.86]	0.09 [−0.45, 0.63]
**BDI-II**												
Total	**−4.01 [−7.42**, **−0.69]**	**−0.61 [−1.15**, **−0.07]**	**−5.72 [−9.13**, **−2.31]**	**−0.87 [−1.43**, **−0.31]**	**−5.68 [−9.17**, **−2.18]**	**−0.86 [−1.42**, **−0.31]**	**−5.68 [−9.17**, **−2.18]**	**−0.86 [−1.42**, **−0.31]**	0.05 [−3.95, 4.05]	0.01 [−0.54, 0.56]	0.05 [−3.95, 4.05]	0.01 [−0.54, 0.56]

*LMM, Linear mixed model; MD, Mean difference; SMD, Standardized mean difference (Hedge's g); IBSSI, Irritable Bowel Syndrome Severity Index; VSI, Vischeral Sensitivity Index; IBS-QOL, Irritable Bowel Syndrome-Quality Of Life. Bold values indicate statistically significant mean differences based on a P < 0.05 level and their 95% confidence intervals*.

The IBSSI-J improved significantly from baseline to mid-treatment, post-treatment, 3-month follow-up, and 6-month follow-up. The effect size of the IBSSI-J was large from baseline to mid-treatment [Hedges' g = −0.98 (−1.54, −0.41)], post-treatment [Hedges' g = −1.48 (−2.09, −0.88)], 3-month follow-up [Hedges' g = −1.78 (−2.41, −1.14)], and 6-month follow-up [Hedges' g = −1.76 (−2.39, −1.13)].

### Secondary Outcome Measures

The VSI improved significantly from baseline to post-treatment, 3-month follow-up, and 6-month follow-up. The effect size of the VSI was large from baseline to post-treatment [Hedges' g = 1.06 (0.49, 1.63)], from baseline to 3-month follow-up [Hedges' g = 1.20 (0.62, 1.78)], and from baseline to 6-month follow-up [Hedges' g = 1.19 (0.61, 1.78)].

The total score of IBS-QOL improved significantly from baseline to mid-treatment, post-treatment, 3-month follow-up, and 6-month follow-up. The effect size of the IBS-QOL was large from baseline to mid-treatment [Hedges' g = 1.04 (0.46, 1.61)], post-treatment [Hedges' g = 1.73 (1.1, 2.36)], 3-month follow-up [Hedges' g = 1.75 (1.12, 2.38)], and 6-month follow-up [Hedges' g = 1.67 (1.05, 2.29)].

The subscales of IBS-QOL, such as dysphoria, interference with activity, body image, health worries, food avoidance, social reaction, and relationships except for sexual concerns, improved significantly from baseline to mid-treatment or post-treatment, 3-month, and 6-month follow-up. The effect size of the subscales of IBS-QOL was medium to large from baseline to post-treatment, 3-month follow-up, and 6-month follow-up (see Table 5, Appendices 1, 2).

In the SF-36 subscales, the effect size of “role physical” was large from baseline to each follow-up, “role emotional” was large from baseline to 6-month follow-up, and “mental health” was medium from baseline to post-treatment and 6-month follow-up (see Table 5).

In STAI, “state anxiety” improved significantly from baseline to mid-treatment, post-treatment, 3-month follow-up, and 6-month follow-up. The effect size of “state anxiety” was large from baseline to post-treatment [Hedges' g = −0.85 (−1.41, −0.29)].

The total score of BDI-II improved significantly from baseline to mid-treatment, post-treatment, 3-month follow-up, and 6-month follow-up. The effect size of the total score of BDI-II was large from baseline to post-treatment [Hedges' g = −0.87 (−1.43, −0.31)], 3-month follow-up [Hedges' g = −0.86 (−1.42, −0.31)] and 6-month follow-up [Hedges' g = −0.86 (−1.42, −0.31)] (see Table 5).

### Responder Status

The responder rate in the IBSSI-J at post-treatment was 42.9% (6/14), at the 3-month follow-up was 53.8% (7/13), and at the 6-month follow-up was 53.8% (7/13). Meanwhile, the responder rate in the VSI at post-treatment was 57.1% (8/14), at the 3-month follow-up was 69.2% (9/13), and at the 6-month follow-up was 69.2% (9/13). The IBS-GIS responder rate in this study was 68.8% at post-treatment, 50% at the 3-month follow-up, and 56.3% at the 6-month follow-up.

### Adverse Events

There were no severe adverse events throughout the interventions.

## Discussion

We developed a hybrid CBT-IE, which we demonstrated to be a safe and feasible intervention method and acceptable treatment for refractory IBS in Japan. Notably, the hybrid CBT-IE induced a statistically significant change in IBSSI-J scores and most of the secondary outcomes, except some subscales of SF-36, with a medium-to-large effect size in patients with IBS. Specifically describing the potential of hybrid CBT-IE, we concluded that the severity of IBS, visceral anxiety, IBS-specific health-related QOL, state anxiety, and depression could improve in the medium to long term. In addition, the dropout rate for failure to complete the 10 sessions was low and no significant adverse events were observed.

Responder rates were comparable to our previous face-to-face only CBT-IE feasibility study. In the previous study (20), participants were diagnosed with IBS by Rome III with moderate to severe symptoms (*N* = 20). In addition, the responder rates in the IBS-GIS were also comparable to our previous face-to-face only feasibility study (20).

The hybrid CBT-IE was not inferior to, less effective, less feasible, or less safe than the face-to-face only CBT-IE. There was no difference in dropout and responder rates in the IBSSI-J and IBS-GIS in all assessment points between the two forms of CBT-IE (21), proving that the hybrid CBT-IE program did not negate the beneficial effects while halving the session time compared to the face-to-face only CBT-IE. In particular, the primary endpoint IBSSI-J decreased with a mean difference of 121.22 (95% confidence interval 68.84–173.61), which was slightly below a change score of 50% as a benchmark of clinical improvement (24). Furthermore, the minimal clinically important difference (MCID) of the total score of the IBS-QOL was between 10 and 14 (33). The mean difference of the IBS-QOL in this study was 26.77 (95% confidence interval 17.49–36.05), which was higher than the value of the MCID of the IBS-QOL. We speculate that the patient's preparation using the video material in advance helped both the patient and therapist focus more on developing the patient's adaptive cognitive behavioral skills in the subsequent face-to-face session. We also found that our hybrid CBT-IE seemed to reduce symptom recurrence more than the face-to-face only CBT-IE in the long-term 6-month follow-up (34).

The beneficial aspects of both CBT-IE and video materials were merged in the hybrid CBT-IE without discarding either. Previous studies have suggested that the presence or absence of therapist direction in CBT sessions is related to effectiveness (35), and this finding is supported by our current results. Hybrid CBT-IE is expected to make it easier for patients to implement *in vivo* exposure as well as IE to situations and sensations they fear and avoid (18, 36, 37); it is often difficult to do so without the direct or indirect guidance of a therapist. In addition, the video materials can be used as a teaching aid for therapists unfamiliar with IBS, which may make it easier for them to implement CBT-IE. Thus, the hybrid CBT-IE can be implemented while retaining the best features of face-to-face only CBT-IE.

We describe the possible future development of CBT for IBS and the hybrid CBT in the post-COVID-19 era. Recently, in the field of CBT for IBS, a randomized controlled trial (RCT) was conducted to assess clinical responses to home-based minimal-contact CBT (MC-CBT) compared with clinic-based standard CBT. MC-CBT minimizes the frequency of visits to medical facilities (38). It consists of psychoeducation, relaxation, cognitive restructuring, problem solving, and relapse prevention, with only four face-to-face meetings with a therapist of 50 min each and home study materials to cover the same procedures as clinic-based-CBT. It has been suggested by Lackner et al. that 10 sessions of clinical-based standard CBT does not appear to provide an incremental advantage over four sessions of home-based CBT, despite a 60% reduction in the time required by clinicians. Meanwhile, it has been noted that MC-CBT has a slower onset of therapeutic effect than standard CBT (39). With the current COVID-19 pandemic, based on the aforementioned advantages and disadvantages of MC-CBT, it may be necessary to study how far we can reduce the number of face-to-face sessions for a hybrid CBT-IE in the future, to the extent that exposure can be properly implemented. In addition, an RCT of group therapy has been performed for CBT-IE in Japan, and the results are awaited (40).

To confirm the effectiveness of the hybrid CBT-IE, we are conducting a multicenter, parallel-design randomized control trial (23). We need to increase the number of participants and investigate the mechanisms of the hybrid CBT-IE, focusing on attentional function, changes in dysfunctional thinking (e.g., catastrophic thoughts), and reducing the use of safety behavior and safety signals included the hybrid CBT-IE.

### Limitations

Four limitations of this study should be noted. First, this study had an open-labeled, single-arm design. The symptom reductions observed in this study are difficult to distinguish from remission seen during normal treatment. However, improvement scores shown in not only the IBSSI-J but also the IBS-QOL had higher values than those in the placebo in the RCT of the drug development (33, 41). Caution must be exercised in interpreting the results due to the small sample size and single-group nature of our study, and further validation is an issue for the future. The hybrid CBT-IE seems to be worth analyzing in an RCT. Second, a single facility participated in this study. It is possible that the results were affected by this design (42). Ideally, therapists from multiple and diverse backgrounds should have participated in the study to disseminate the hybrid CBT-IE widely with the aim of achieving a certain level of effectiveness regardless of which therapist implemented it. Third, we did not examine participants' learning effect to check whether the follow-up results were influenced by reviewing the video materials and other materials after the intervention was completed. We suggest that the learning effect using psychoeducational materials needs to be examined. Fourth, this study was not originally designed as a non-inferiority study, and thus, the results should be interpreted with caution.

## Conclusions

This study examined the feasibility and efficacy of the hybrid CBT-IE for refractory IBS in Japan. The results indicated that the dropout rate in this study was comparable to our previous face-to-face only CBT-IE. It was also suggested that the hybrid CBT-IE was effective and could be conducted safely; it is potentially effective for improving IBS severity, visceral anxiety, and QOL.

## Data Availability Statement

The raw data supporting the conclusions of this article are available from the corresponding author upon reasonable request.

## Ethics Statement

The studies involving human participants were reviewed and approved by Ethics Committee in National Center of Neurology and Psychiatry, Tokyo, Japan. The patients/participants provided their written informed consent to participate in this study.

## Author Contributions

TA was the primary investigator and conducted this study. TA, YF, HK, CO, and NS designed this study. YO, SK, MH, and TA developed the Japanese version of CBT-IE and its psychoeducational materials. YF and TA developed the video materials. TA, YT, and HA recruited and screened participants. HitK and MF conducted the interventions and corrected data. KH did data entry. MF and KM analyzed the data. TA, YF, AS, and SF supervised the overall conduct of the study. The initial draft manuscript was written by MF. All authors read and approved the final manuscript to be published.

## Funding

This study was funded by Intramural Research Grant (29-2) for Neurological and Psychiatric Disorders of NCNP (to TA); a Grant-in-Aid for Scientific Research from the MHLW (H29-Nanbyou-Ippan-059) from The Nakatomi Foundation (to AS); JSPS KAKENHI Grant-in-Aid for Scientific Research (C) 19K07882 (to MF, TA, and HitK); and a grant from The Mental Health Okamoto Memorial Foundation (to MF).

## Conflict of Interest

The authors declare that the research was conducted in the absence of any commercial or financial relationships that could be construed as a potential conflict of interest.

## Publisher's Note

All claims expressed in this article are solely those of the authors and do not necessarily represent those of their affiliated organizations, or those of the publisher, the editors and the reviewers. Any product that may be evaluated in this article, or claim that may be made by its manufacturer, is not guaranteed or endorsed by the publisher.

## Footnotes

^1^ https://www.ncbi.nlm.nih.gov/pubmed/21656972

## Supplementary Material

Appendix 1 Inference results for fixed effects LMM.

Appendix 2 Inference results for covariance parameters LMM.

## Abbreviations

IBS: irritable bowel syndrome
CBT: cognitive behavioral therapy
CBT-IE: cognitive behavioral therapy using interoceptive exposure
AC: attention control
QOL: quality of life
M.I.N.I.: Mini-International Neuropsychiatric Interview
LMM: linear mixed model
RCT: randomized control trial.
